# Interactions between dense seasonal macroalgal mats and oysters on natural and constructed shellfish reefs

**DOI:** 10.7717/peerj.20682

**Published:** 2026-02-12

**Authors:** Carter S. Smith, Michelle C. Brodeur, Stephanie R. Valdez, F. Joel Fodrie, Y. Stacy Zhang

**Affiliations:** 1 School of Aquatic and Fishery Sciences, University of Washington, Seattle, WA, United States; 2 Nicholas School of the Environment, Duke University, Beaufort, NC, United States; 3 North Carolina Division of Marine Fisheries, Morehead City, NC, United States; 4 Institute of Marine Sciences, University of North Carolina at Chapel Hill, Morehead City, NC, United States; 5 Department of Marine, Earth and Atmospheric Sciences, North Carolina State University, Raleigh, NC, United States

**Keywords:** Oyster, Macroalgae, Algal mat, Drift algae, Facilitation, Restoration, *Crassostrea virginica*

## Abstract

Oysters are important coastal foundation species that provide valuable hard substrate for the recruitment of epibiotic organisms in environments otherwise dominated by soft sediment. Yet, their interactions with epibionts are relatively understudied. Despite the proliferation of macroalgal mats across the Southeastern United States in winter months, the relationship between oysters (*Crassostrea virginica*) and seasonal macroalgae is poorly understood. We conducted an observational field survey and two manipulative field experiments to document seasonal macroalgal dynamics on intertidal oyster reefs and to better understand the interaction between the oysters and algae. We found that algal mats in North Carolina were primarily composed of two genera, *Ulva* and *Ectocarpus*, which together reached extremely high cover (up to 100%) over large areas of reef. Macroalgae appeared in January and declined in May, with peak cover in February and March. Algal cover was significantly higher on constructed oyster reefs *vs*. natural oyster reefs. Our field experiments showed that algal cover was significantly higher on dead oyster mimics *vs*. live oysters, suggesting that the primary mechanism of algal facilitation is associated with the provisioning of hard substrate rather than fertilization. Reciprocally, we found no significant effects of macroalgae on oyster abundance or growth, likely due to relatively low algal cover in the experimental treatments. With a predicted proliferation of macroalgae under global change, our study highlights the important role that intertidal oyster reefs play in providing substrate for macroalgae, but more research on this key species interaction in intertidal areas of the Southeastern United States is needed.

## Introduction

Interactions between species are of fundamental importance (Bertness & Callaway, 1994; Hardin, 1960; Paine, 1966), as they influence the structure and functioning of ecosystems (Pimm, 1984). Foundation species, such as trees in terrestrial systems and corals in marine systems, play a critical role in ecosystem development by providing the physical habitat and resources needed for organisms to survive (Bruno, Stachowicz & Bertness, 2003). Nevertheless, the strength and direction of species interactions can be highly context-dependent (Bertness & Callaway, 1994; Chamberlain, Bronstein & Rudgers, 2014) and are likely to shift under global change (He, Bertness & Altieri, 2013). Therefore, detailed knowledge of key species interactions in foundational habitats is crucial for basic understanding and for predicting the stability and resilience of ecosystems in the future.

Oysters are an important coastal foundation species that facilitate biodiversity. In some areas, such as the East and Gulf coasts of the USA, oyster shell is one of few naturally occurring hard substrates that is available for the recruitment of epifaunal organisms like barnacles, polychaetes, bryozoans, and macroalgae (Glenn et al., 2020; Lenihan, 1999, Wells, 1961). Moreover, oysters may also facilitate epiphytes *via* improved water quality from filter feeding (Newell & Koch, 2004) and fertilization from releasing nutrient-rich pseudofeces (Locher et al., 2021). While some colonizing epibionts can have reciprocal positive impacts on foundation species and overall biodiversity (*e.g*., secondary foundation species; Thomsen et al., 2018), others can be detrimental to foundation species survival and growth (*e.g*., heavy epiphytic algae loads can reduce seagrass photosynthesis; Van Montfrans, Wetzel & Orth, 1984). On oyster reefs, fouling of subtidal oysters by filter feeders has been shown to strongly reduce growth (Bishop & Peterson, 2006), and experimental additions of drift algae have been shown to reduce oyster recruitment (Thomsen & McGlathery, 2006). In contrast, green algae growing with oysters has been shown to mitigate the negative effects of ocean acidification on oysters (Young & Gobler, 2018), and macroalgae could benefit oysters in the intertidal by retaining moisture and lowering desiccation stress (similar to algae in the rocky intertidal; Leonard, 1999).

Intertidal oyster reefs are subject to strong abiotic gradients that may influence the magnitude and directionality of species interactions across small spatial and temporal scales. For example, the Stress Gradient Hypothesis (Bertness & Callaway, 1994) predicts that stress ameliorating positive interactions between species will be more likely in abiotically stressful environments (*e.g*., the high intertidal) whereas negative interactions like competition and predation are more likely in more benign environments (*e.g*., the low intertidal). In fact, work by Fodrie et al. (2014) showed that intertidal competition and predation significantly impacted oyster survival at lower tidal elevations. Moreover, seasonal dynamics such as changes in temperature, nutrients, and the life history patterns of species can shift ecological relationships over the course of a year (Bloomfield et al., 2022). Therefore, a better understanding of the interactions between intertidal oysters and their epibionts across space and time is needed to improve our understanding of the basic ecology of this important coastal system and to better predict how oyster reefs can be conserved and restored under global change.

In this study, we investigate a conspicuous and widespread, but poorly understood, phenomenon—the formation of seasonal macroalgal mats on intertidal *Crassostrea virginica* oyster reefs along the Southeastern USA coast. First, we conducted an observational field survey in 2012 to explore the phenology of macroalgal recruitment on oyster reefs and to determine if there were any differences in macroalgal cover on natural *vs*. restored reefs. Restored reefs are typically younger and can have different characteristics than natural reefs (*e.g*., higher oyster abundance; Grabowski et al., 2022) and we wanted to understand whether restoration context could affect algal abundance. Second, to gain a mechanistic understanding of the interactions between oysters and algae, we conducted two field experiments to explore the following hypotheses: (1) Live *C. virginica* provide the best recruitment substrate for seasonal macroalgae *via* substrate and nutrient deposits; and, (2) the effects of macroalgae on oysters vary across an intertidal stress gradient, with positive effects in the higher intertidal and negative effects in the lower intertidal.

## Materials and Methods

### Field site description

**Figure 1:**
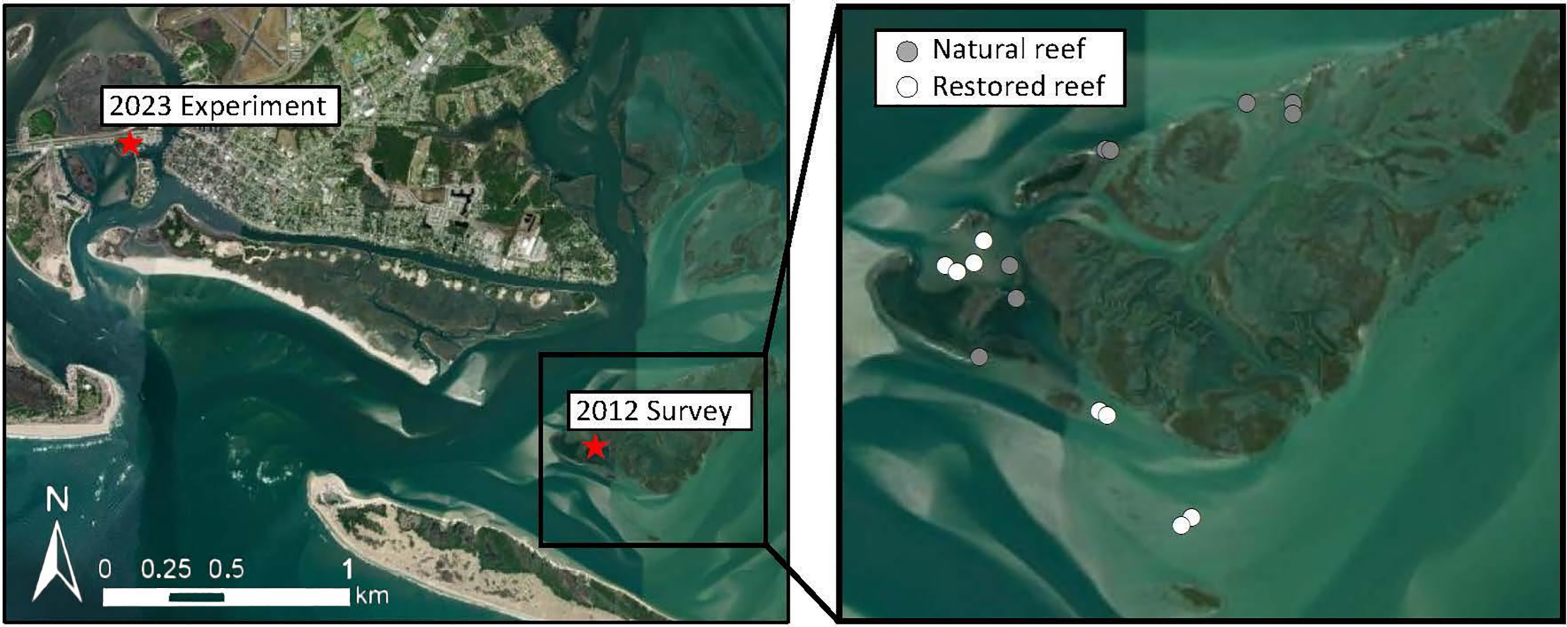
Map of study area. Shows the locations of the 2012 observational surveys and the 2023 field experiments.

In early 2012, we quantified seasonal dynamics of macroalgal cover on natural and restored intertidal oyster reefs in North Carolina, USA. We selected eight natural reefs and eight restored reefs (restored between 1997–2000) within Middle Marsh (34°41′32.6184″N, −76°37′37.92″W), which is part of the Rachel Carson National Estuarine Research Reserve (NERR; NC Coastal Reserve Research Permit #16-2010; Fig. 1). Middle Marsh is a mosaic of marsh islands, tidal flats, seagrass meadows, and natural and restored oyster reefs. Additionally, in the winter of 2023, we conducted two field experiments near Pivers Island, NC (34°43′17.76″N, −76°40′25.9314″W) located adjacent to the Rachel Carson NERR and approximately 6 km from Middle Marsh (Fig. 1). Both experiments were conducted in the tidal flat adjacent to a natural marsh shoreline. All field sites were located within Back Sound, which is a protected saltwater sound with a mean salinity of 35 ppt and tidal range of approximately 1 m.

### Field survey 2012

At each of the 16 reefs selected for our observational field survey (eight natural and eight restored), we established a 0.25 m^2^ permanent monitoring plot in both the high and low intertidal zone (high-elevation plots were marked at −0.27 ± 0.03 m (mean ± SE) NAVD88 and low-elevation plots were marked at −0.40 ± 0.02 m). Each plot was visited at low tide, approximately every 2 weeks from January to May, and we visually estimated total percent cover of macroalgae.

### Field experiment 2023

**Figure 2:**
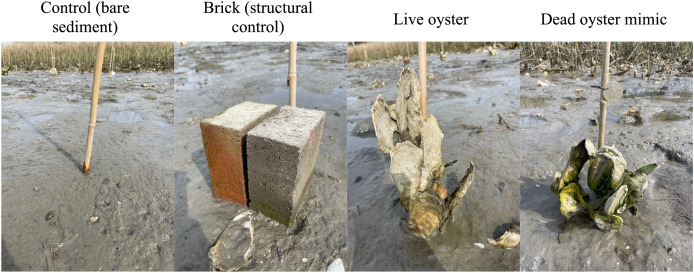
Photographs of the experimental treatments used in the 2023 field experiment.

To quantify the recruitment of macroalgae on oyster reefs and assess any reciprocal effects of macroalgae on oyster growth, we conducted two complementary field experiments. First, to determine whether live oysters provide the best substrate for macroalgal recruitment, we conducted a fully crossed 2-factor experiment manipulating tidal elevation (two levels: high and low) and substrate type (four levels: control (*i.e*., bare sediment), structural control (*i.e*., two red-clay bricks place side-by-side), live oyster clumps, and dead oyster clump mimics)) (*n* = 10; Fig. 2). To prepare the live and dead oyster treatments, we collected live oyster clumps from the same location as the field experiment (with an average of 14 ± 3 live oysters per clump, and an average displacement volume of 5 ± 1 L). The clumps were scrubbed and cleaned to remove all fouling organisms and randomly assigned to one of two substrate treatments: live oysters or dead oysters. Live oyster clumps were maintained as they were in flow through seawater tanks; while dead oyster treatments were placed in a drying oven for 1.25 h at 200 °C to kill all oysters and cooled to 60 °C for 24 h. All dead oyster tissue was then manually removed, and z-spar marine epoxy was used to reseal the oyster shells and maintain the structure of the oyster clump (Fig. 2). The treatments were deployed in early January 2023 near the Rachel Carson NERR (Fig. 1), by randomly assigning treatments to different experimental plots along an intertidal shoreline. The plots were established at two different elevations in the low intertidal zone (higher elevation plots were marked at −0.46 ± 0.02 m NAVD88 and lower elevation plots were marked at −0.54 ± 0.02 m). We worked exclusively in the low intertidal zone for this experiment based on the results from the observational survey and the expectation that we would have higher macroalgal cover and greater power to detect differences among treatments. Experimental treatments were deployed in the field by inserting the base of the oyster clumps (or the base of the bricks) into the sediment and zip-tying the clumps to a bamboo stake in each of the plots. All treatments were left undisturbed until March (during peak algal biomass) at which point we quantified total macroalgal cover on each treatment using the point-intercept method (*sensu* Floyd & Anderson, 1987).

To test the effects of macroalgal growth and tidal elevation on oyster growth, we ran an additional 2-factor experiment manipulating tidal elevation (two levels: higher and lower low intertidal, as above) and macroalgal cover (two levels: ambient macroalgal growth and macroalgal removal) (*n* = 10). As above, we collected live oyster clumps from the location of the field experiment. We counted the total number of oysters on each clump, and four oysters on each clump were marked with Betterbee tags and measured for initial size (*i.e*., height × width). Oyster clumps were randomly allocated to a treatment and deployed in the field as above in early January 2023. Live oyster clumps with ambient macroalgal growth were the same as the live oyster clump treatments in the first experiment and they were left undisturbed for the duration of the field experiment. Oyster clumps in the macroalgal removal treatment were scrubbed biweekly with a toothbrush to remove any new macroalgae. All treatments were recovered from the field in March, at which point we brought the oyster clumps back to the lab, counted the total number of live oysters, and identified and remeasured live tagged oysters to assess growth. We recovered 79% of the oysters that were originally tagged (including oysters that were alive or dead), with at least one oyster recovered from each clump. We averaged across all tagged oysters on a clump to get one estimate of oyster growth per clump (the average standard error for intra-clump variability in growth was 39 mm^2^).

### Data analysis

Data from the 2012 observational field survey of total macroalgal cover on oyster reefs was analyzed using a generalized linear mixed effects model with an ordered beta distribution in the ‘glmmTBM’ package in R (Brooks et al., 2017). Treatment (categorical with two levels: natural and restored), elevation (categorical with two levels: low and high intertidal), and month (categorical with five levels: January, February, March, April, and May) were included as fixed effects, and reef was included as a random effect to account for repeated measurements over time. We used Akaike’s Information Criterion (AIC) to select the most parsimonious model (*i.e*., the model with the lowest AIC), from a suite of models that included the fully saturated model all the way down to the intercept only model. For transparency, we also report when two models were similar (*i.e*., ΔAIC < 2), but results are only presented for the best model. With the final model, we used the ANOVA function in the ‘car’ package (Fox & Weisberg, 2019) to get *p*-values for each of the model terms.

Percent cover data from the first field experiment, collected *via* the point-intercept method, were analyzed using a generalized linear model with a binomial distribution and the model was weighted by the total number of intercept points. Treatment (categorical with four levels: control, brick, live oyster, dead oyster) and elevation (categorical with two levels: low and high low-intertidal) were included as fixed effects. As above, we used AIC to select the best model and the ‘car’ package to calculate *p*-values. We also used the ‘emmeans’ package (Lenth, 2023) to get pairwise differences among treatments.

The change in the total number of live oysters and the change in the growth of tagged oysters from the second experiment, were analyzed using two-way ANOVA with treatment (categorical with two levels: ambient macroalgae and macroalgal removal) and elevation (two levels: low and high low-intertidal) included as fixed effects as well as their interaction. All analyses were conducted in R (version 4.4.1; R Core Team, 2024).

## Results

### Field survey 2012

**Figure 3:**
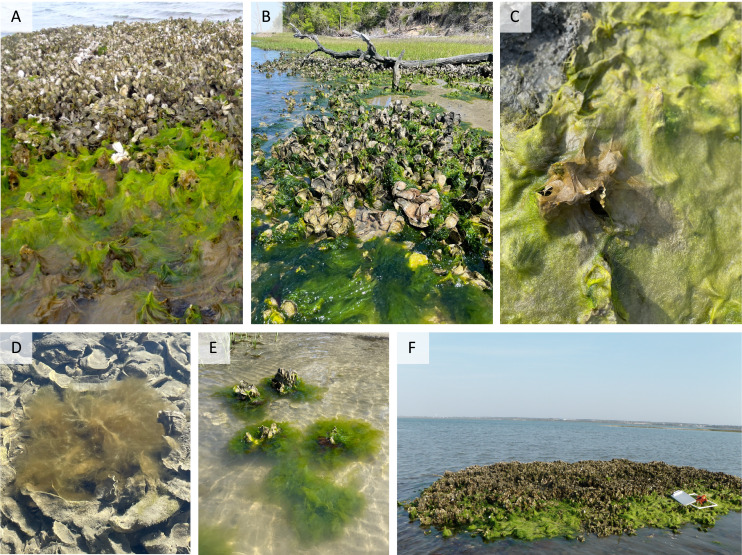
Photographs of seasonal macroalgal mats on oyster reefs in North Carolina. (A) Emergent small-diameter proliferously branched tube-form *Ulva* sp. with *Ectocarpus* sp. on a natural oyster reef (low tide, Middle Marsh, NC on March 14th, 2011); (B) blade-form *Ulva* sp. on a natural oyster reef (mid tide, Pine Knoll Shores, NC on April 19th, 2023); (C) emergent small-diameter proliferously branched tube-form *Ulva* sp. with *Ectocarpus* sp. (on the center oysters that are vertically protruding) on a natural oyster reef (low tide, Beaufort, NC on February 20, 2023); (D) submerged *Ectocarpus siliculosus* on a natural oyster reef (mid tide, Beaufort, NC on February 7th, 2024); (E) submerged blade-form *Ulva* sp. on natural oyster clumps (mid tide, Pine Knoll Shores, NC on April 19th, 2023), (F) emergent small-diameter proliferously branched tube-form *Ulva* sp. on a restored oyster reef (low tide, Middle Marsh, NC on March 29th, 2012) (Photo credits: Michelle Brodeur (A and F) and Carter Smith (B, C, D, and E)).

**Figure 4:**
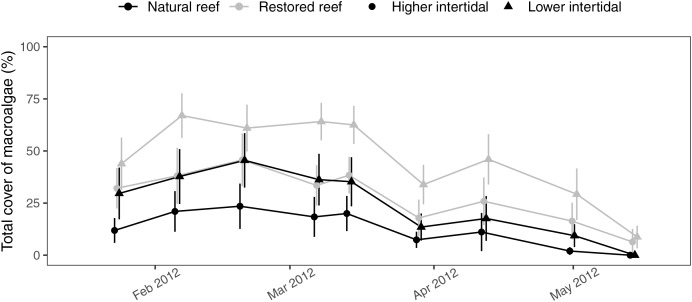
Plot showing the total macroalgal percent cover over time on natural and restored oyster reefs from the observational survey conducted in 2012. All reefs were sampled on the same days, but the points in the graph have been slightly offset for easier viewing. Points show mean ± se (*n* = 8).

Over the 5-month field survey, we found that macroalgal mats reached extremely high cover on oyster reefs during winter months (*i.e*., 100% cover in some plots), while mats were virtually absent on surrounding low-structural-complexity mudflats (M. Brodeur, 2012, personal observation). These algal mats were predominantly composed of two genera, *Ulva* and *Ectocarpus* (*i.e*., >95% combined cover; Fig. 3). Most algae were observed to be directly attached to the underlying oysters (*i.e*., algae did not drift in from another location). The most parsimonious model for macroalgal cover contained all fixed effects and no interactions (but alternative models that included interaction terms between treatment and elevation and treatment and month were not meaningfully different (ΔAIC < 2)). Macroalgal cover varied by month (generalized linear mixed model, χ^2^ = 112.4, *p* < 0.001), with peak cover from February to March that subsequently declined through May (Fig. 4). Macroalgal cover was higher in the low-intertidal *vs*. the high-intertidal (χ^2^ = 46.4, *p* < 0.001), with an average cover of 36 ± 3% in the low-intertidal and 21 ± 2% in the high-intertidal across all months. Cover was also significantly higher on restored *vs*. natural oyster reefs (χ^2^ = 4.6, *p* = 0.03), with average macroalgal cover in the low intertidal peaking at 67 ± 11% on restored reefs and 46 ± 13% on natural reefs (Fig. 4; Table 1). Note that residuals were mildly non-uniform and therefore coefficient estimates should be interpreted with this in mind.

**Table 1:** Generalized linear mixed model results from analysis of the 2012 observational data on total macroalgal cover (%). Month (January–May), elevation (higher and lower intertidal), and treatment (restored and natural reef) were included as fixed effects, and reef was included as a random effect to account for repeated measurements over time at the same sites.

	Total macroalgal cover		
*Predictors*	*Estimates*	*std. Error*	*95% CI*
(Intercept)	−2.04	0.40	[−2.83 to −1.26]
Month (February)	0.80	0.20	[0.40–1.20]
Month (March)	0.29	0.19	[−0.09 to 0.67]
Month (April)	−0.08	0.24	[−0.55 to 0.39]
Month (May)	−1.46	0.25	[−1.95 to −0.96]
Elevation (Low)	0.81	0.12	[0.59–1.04]
Treatment (Restored)	1.14	0.50	[0.16–2.12]
**Random effects**			
σ^2^	0.33		
τ_00__reef_	0.93		
ICC	0.74		
N _reef_	16		
Observations	288		
Marginal R^2^/Conditional R^2^	0.473/0.863		

### Field experiments 2023

**Figure 5:**
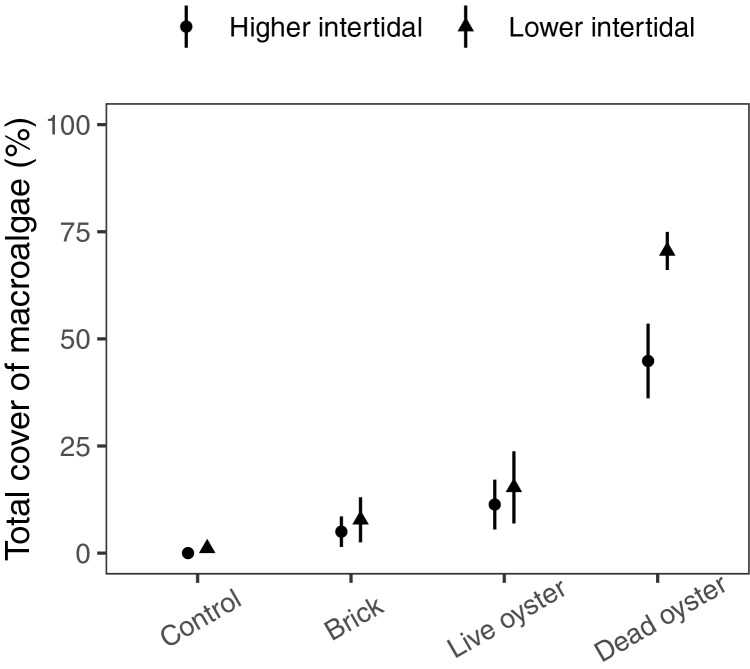
Plot showing macroalgal cover on different experimental treatments in the 2023 field experiment. Points show mean ± se (*n* = 10).

Macroalgal cover on experimental treatments was dominated by *Ulva* spp., which was predominantly growing on (*i.e*., directly attached to the provided substrate), rather than just entangled with, each of the structural treatments. The most parsimonious model for macroalgal cover contained both fixed effects (elevation and treatment) and no interaction. Cover was significantly higher in the low-elevation plots (generalized linear model, χ^2^ = −5.8, *p* = 0.02), and significantly different among treatments (χ^2^ = −139.5, *p* < 0.001; Table 2). Algal cover in the bare sediment treatment was virtually nonexistent and significantly lower than the live and dead oyster treatments (adjusted *p* < 0.05). Cover was highest in the dead oyster treatment *vs*. all other treatments, including live oyster (adjusted *p* < 0.001 for all dead oyster treatment contrasts; Fig. 5; Table 3).

**Table 2:** Generalized Linear Model results from analysis of the 2023 experimental data on total macroalgal cover (%). Treatment (control, brick, dead oyster, and live oyster) and elevation (high and low low-intertidal) were included as fixed effects.

	Total macroalgal cover		
*Contrast*	*Log-Odds*	*Std. Error*	*95% CI*
(Intercept)	−5.63	1.03	[−8.52 to −4.07]
Treatment (Brick)	2.63	1.06	[0.94–5.55]
Treatment (Dead oyster)	5.52	1.03	[3.95–8.41]
Treatment (Live oyster)	3.45	1.04	[1.82–6.36]
Elevation (Low)	0.75	0.32	[0.14–1.38]
Observations	80		
R^2^ Tjur	0.039		

**Table 3:** Results of the pairwise comparison of different treatments (control, brick, dead oyster, and live oyster) in the analysis of the 2023 experimental data on total macroalgal cover (%). See Table 2 for the full model results.

	Total macroalgal cover		
*Predictors*	*Estimate*	*Std. Error*	*adj. p-value*
Control–Brick	−2.6	1.06	0.06
Control–Dead oyster	−5.5	1.03	<0.001
Control–Live oyster	−3.5	1.04	<0.01
Brick–Dead oyster	−2.9	0.42	<0.001
Brick–Live oyster	−0.8	0.45	0.27
Dead oyster–Live oyster	2.1	0.37	<0.001

**Figure 6:**
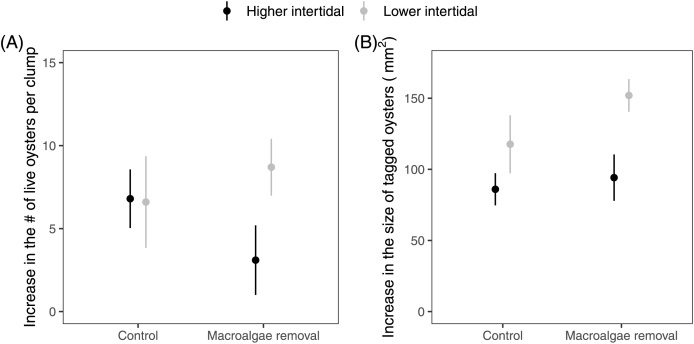
Plots showing the change in live oyster abundance and size from the beginning to the end of the 2023 field experiment. (A) The total increase in live oyster abundance on oyster clumps and (B) the total increase in the size of tagged oysters that were alive at the end of the experiment. Points show mean ± se (*n* = 10).

There was no statistically detectable difference in the change in the abundance of live oysters on oyster clumps in relation to either elevation (Two-way ANOVA; F_1,36_ = 1.62, *p* = 0.21), macroalgal presence (F_1,36_ = 0.14, *p* = 0.71), or their interaction (F_1,36_ = 1.86, *p* = 0.18; Fig. 6A). Elevation had a statistically significant impact on the change in the size of tagged oysters that were alive at the end of the experiment, with oysters growing significantly more in the lower intertidal (F_1,36_ = 8.51, *p* = 0.006). Oysters also trended towards higher growth when macroalgae was removed, but this effect was not statistically significant (F_1,36_ = 1.92, *p* = 0.174). The interaction between elevation and macroalgal presence was not statistically significant (F_1,36_ = 0.73, *p* = 0.40; Fig. 6B).

## Discussion

In this study, we document the formation of seasonal macroalgal mats on intertidal *C. virginica* oyster reefs in North Carolina, USA. Across our observational survey and field experiments, we found high cover of macroalgae attached to natural, restored, and experimental oysters in winter and spring months. Epiphytic algae have been documented growing on oyster reefs previously (Andriana, van der Ouderaa & Eriksson, 2020; Glenn et al., 2020; Connor, 1980; Fodrie et al., 2014; Rhodes, 1970) but not to the extent observed in this study (*i.e*., nearly 100% coverage over some areas of reef). Our results suggest that oysters provide a valuable hard substrate for the direct recruitment of algae, and reciprocally, that macroalgae have minimal effects on oyster growth. Ultimately, this suggests that the interaction between oysters and macroalgae can be facilitative, but further work is needed to understand the conditions under which each species group may inhibit the other.

Bivalves can provide a crucial hard substrate for algal attachment (Zhang & Silliman, 2019) and their waste products can also act as a fertilizer that promotes primary production (Hargrave et al., 2021). A recent meta-analysis by Clemente & Thomsen (2023) found that bivalves tended to have an overall positive effect on seaweeds, but that the interactions between bivalves and seaweeds were generally understudied. Moreover, *Ulva*, which was one of the most common algal groups in our plots (and has been observed frequently colonizing other oyster reef ecosystems; Glenn et al., 2020), has been shown to attach weakly to substrate and likely has high breakage and thus transport to other systems (Thomsen, 2004). The fact that the algae were directly attached to underlying oysters, rather than entangled with them, suggests that oyster reefs may be an important source of drift algae to other coastal ecosystems (*e.g*., salt marshes and seagrass beds where drift algae often become entangled and accumulate high biomass).

We observed higher macroalgal cover on restored *vs*. natural oyster reefs. Previous research has shown that restored ecosystems often have different configurations and support different communities than natural reference systems (Nero, 2021), which has implications for the overall functioning of restored systems. In a study of many of the same reefs that we sampled, Grabowski et al. (2022) found that restored reefs had higher oyster cluster weight and higher densities of live adult oysters than natural reference reefs, resulting in higher structural complexity on restored reefs. While we did not measure structural complexity or live oyster densities on the reefs in our study, it is possible that higher structural complexity on restored reefs provides more abundant attachment substrate for algae or that higher densities of live oysters provide higher levels of fertilization for the algae. Moreover, research by Glenn et al. (2020) found that algal biomass and richness were similar on natural oyster reefs *vs*. oyster farm gear, but that community composition differed and there was a much higher proportion of non-native species on farm gear. Accordingly, future work should evaluate the relative occurrence of native and non-native algal species on restored and natural reefs.

Similar to our field survey, our field experiment found that oysters (both alive and dead) provided valuable substrate for the recruitment of seasonal algae *vs*. bare sediment alone. Nevertheless, contrary to our expectations, we found that algal recruitment was highest in the dead oyster treatment, rather than the live oyster treatment, suggesting that the primary mechanism of facilitation is the provisioning of substrate rather than fertilization. Previous research suggests that bivalves may consume macroalgal propagules (Peterson & Heck, 2001; Smith et al., 2018) and therefore live oysters may reduce macroalgal recruitment *via* consumption. For example, Kasim & Mukai (2009) found that oysters can preferentially feed on different kinds of epiphytic microalgae, though it is not clear if oysters also eat epiphytic macroalgal propagules. More research is needed to investigate the interaction between live *vs*. dead oyster density and algal recruitment. In many locations, successful oyster restoration requires the addition of hard substrate (often in the form of loose oyster shell) for larval oysters to recruit to and establish. Early in the restoration trajectory of these reefs, shell cover is high, but live oyster cover is typically low; based on our results, this high proportion of dead shell may facilitate higher algal recruitment and biomass than on natural reefs. Previous studies have shown that algae can be detrimental to oysters when at high biomass (Bishop & Peterson, 2006 and Thomsen & McGlathery, 2006), and therefore this could ultimately slow or compromise reef restoration in areas where there is high seasonal overlap in peak algal biomass and oyster recruitment.

We did not find any support for our hypothesis that the effects of macroalgae on oysters would vary across an intertidal stress gradient, and we found that the effects of elevation were more important than the effects of algae on the oysters. A meta-analysis by Clemente & Thomsen (2023) generally found neutral or slightly positive effects of algae on bivalves, but they noted that many of the interactions were highly context-dependent (*e.g*., by species, algal biomass, and flow intensity). Relatedly, Kochmann & Crowe (2014) found that macroalgae enhanced the growth of Pacific oysters at one site, but reduced growth at another. We saw a weak but non-significant trend of lower oyster growth in algal treatments, but algal cover in our field experiment was generally quite low (*i.e*., <25% on average for live oysters) and may not have been high enough to limit water flow and the delivery of food for the oysters. Moreover, algal cover in the experimental treatments was dominated by *Ulva*, which due to its morphology, may be less likely than *Ectocarpus* to have a smothering effect on the oysters. In fact, in an aquaculture context, Grenn (2025) found that *Ulva* could potentially benefit oysters by improving water column DO and pH. Nevertheless, previous research in soft sediment and coral reef ecosystems has shown that algal mats can increase sedimentation rates, hypoxia, and acidity of the underlying water (Hull, 1987; Martinez, Smith & Richmond, 2012), but this only happens at high algal biomass. Similarly, Thomsen & McGlathery (2006) found that experimentally placed drift algae strongly inhibited *C. virginica* oyster recruitment likely due to smothering and interference with feeding. They tested this effect with high algal biomass in spring and summer months, which coincides with the peak larval recruitment window for oysters (Francis, Heffernan & Walker, 1995; Kelly et al., 2011). In contrast, we observed that high algal biomass growing directly on oysters dissipates by the spring, and therefore the periods of highest algal biomass do not coincide with peak oyster recruitment in North Carolina (Francis, Heffernan & Walker, 1995; Kelly et al., 2011). This asynchrony may result in a limited effect of algae on oyster recruitment. More research is needed to understand how algal biomass and cover mediate the effects of macroalgae on oysters across all four seasons, and to understand the overlap in algal distribution and the optimal growth zone of oysters (Ridge et al., 2015).

Coastal eutrophication is changing algal dynamics in coastal areas all over the world (Ye et al., 2011). Teichberg et al. (2010) found that nitrogen and phosphorus enrichment in seven different coastal systems significantly enhanced the growth of *Ulva* spp. Similarly, experimental work conducted in North Carolina by Brodeur (2016) found that nutrient enrichment could, in some contexts, enhance *Ulva* cover and delay the decline in algal cover until further into the summer season. Altogether, changes in the biomass or timing of macroalgal proliferation under enhanced nutrient conditions could have consequences for oyster recruitment and growth, especially if peak algal biomass overlaps more regularly with peak oyster recruitment season. Ultimately, more work is needed to understand the context-dependent interactions between seasonal macroalgae and natural and constructed oyster reefs, to predict how they will change in the future. Macroalgae have the potential to serve as an important secondary foundation species on oyster reefs, facilitating greater biodiversity than just oysters alone (Thomsen et al., 2018), but high-density macroalgae may inhibit oyster settlement, restrict water flow, and create hypoxic conditions. Therefore, biodiversity may be enhanced by the presence of macroalgae up until a threshold density, above which macroalgae could compromise the survival of the primary foundation species (*i.e*., oysters) and ultimately lower biodiversity.

## Additional Information and Declarations

### Field Study Permissions

The following information was supplied relating to field study approvals (*i.e*., approving body and any reference numbers):

NC Coastal Reserve Research Permit #16-2010.

### Data Availability

The following information was supplied regarding data availability:

All raw data and code needed to replicate analyses is available at Zenodo: Smith, C. (2025). Interactions between dense seasonal macroalgal mats and oysters on natural and constructed shellfish reefs [Data set]. Zenodo. https://doi.org/10.5281/zenodo.17547196.
